# The *Individual- and Organization-Related Stressors in Pandemic Scale for Healthcare Workers* (IOSPS-HW): Development and Psychometric Properties of a New Instrument to Assess Individual and Organizational Stress Factors in Periods of Pandemics

**DOI:** 10.3390/ijerph20054082

**Published:** 2023-02-24

**Authors:** Caterina Primi, Monica Giuli, Emanuele Baroni, Vanessa Zurkirch, Matteo Galanti, Laura Belloni, Costanza Gori, Maria Anna Donati

**Affiliations:** 1 NEUROFARBA (Neuroscience, Psychology, Drug Research and Child Health) Department, University of Florence, 50139 Firenze, Italy; 2 Clinical Operating Unit of the Organizations, Careggi University Hospital, Regional Reference Center on Relational Criticalities (RCRC), 50141 Florence, Italy

**Keywords:** IOSPS-HW, stress, health workers, pandemics, invariance, validity

## Abstract

The validation and psychometric properties of the *Individual and Organization related Stressors in Pandemic Scale for Healthcare Workers* (IOSPS-HW) were presented. This is a new measure to assess individual factors related to the health and well-being of individuals, such as family and personal relationships, as well as organizational factors related to the management of the pandemic, including workplace relationships, job management and communication. Across two studies conducted at different time points of the pandemic, psychometric evidence of the IOSPS-HW is presented. In Study 1, through a cross-sectional design, we conducted exploratory and confirmatory factor analysis through which the originally developed 43 items scale was reduced to a 20-item bidimensional scale with two correlated dimensions: Organization-related Stressors (O-S; 12 items) and Individual- and Health-related Stressors (IH-S; 8 items). Internal consistency and criterion validity were also provided by investigating the relationship with post-traumatic stress. In Study 2, we provided evidence for the temporal invariance of the measure and for temporal stability through a Multigroup-CFA through a longitudinal design. We also supported the criterion and predictive validity. The results suggest that IOSPS-HW is a good instrument to simultaneously investigating individual and organizational factors related to sanitary emergencies in healthcare workers.

## 1. Introduction

Stress can be defined as physiological or psychological change that forces the person to deviate from his normal functioning [1]. This change is caused by the perception of the subject regarding environmental demands [2]. When biological, social or psychological conditions of the environment are perceived to be greater than the available resources, a stressful situation arises [3]. Similarly, work-related stress corresponds to the set of physical and emotional responses due to the inability to face the demands of one’s work [4]. Stress at work is an increasingly common occurrence in modern life and can emerge from a variety of sources, affecting people in different ways [5,6]. Psychosocial stressors in the workplace may occur due to the characteristics of the role, as well as the lack of control and social support. The interaction of these elements can result in harmful effects on the health of individuals and organizations [7,8]. Burnout is one of the multiple common outcomes of chronic work stress, and it is defined as a persistent negative mental state characterized by physical and emotional exhaustion [9]. Employees who experience a high level of work stress have an even greater likelihood of experiencing health problems, poor motivation, less productivity and a sense of occupational safety [10]. Organizations with such employees are less likely to be successful in a competitive market [11].

Each work occupation is characterized by various types and loads of stress. However, Selye [12] indicated that healthcare is one of the most stressful professions. The need to consider and investigate occupational stress in health contexts has been emphasized, as performance strongly decreases in stressful situations [11]. It has been found that stress contributes to organizational inefficiency, high staff turnover, absenteeism due to illness, a decrease in quality and the amount of care, an increase in healthcare costs and a decrease in job satisfaction [11]. The risk of developing health problems for health professionals is 10% higher than the average for other professions, indicating the presence of additional sources of stress [13]. The COVID-19 pandemic has proved to be stressful for people in the community and healthcare professionals. According to the studies conducted on past outbreaks of SARS (Severe Acute Respiratory Syndrome) and MERS (Syndrome Respiratory System of the Middle East), frontline health workers report high levels of stress that often results in depression, anxiety and post-traumatic stress disorder (PTSD) [14,15]. Italy was the first Western country to report cases of COVID-19, and in February 2020, the government declared a state of emergency [16]. During the peak of cases produced by the first wave, many of the major Italian cities suffered overloads of intensive care [17]. Thus, Italian authorities announced strict quarantines along with a country-wide lockdown. Despite all the efforts made by government authorities and the health system, Italy, as well as many other countries, was not ready to face such a pandemic [18]. Healthcare professionals worked continuously at the forefront of the treatment of infected patients, exposing them to critical situations every day [19]. The risk of infection and transmission of the virus to other patients and families was very high, sometimes making it necessary to isolate themselves [20]. The lack of adequate personal devices of protection was another factor that increased concern and the sense of insecurity at work [21]. In an unprecedented situation such as this one, health professionals were certainly the most exposed category. About 86% of the interviewed operators reported increased perceived work stress. Symptoms of anxiety, depression and acute stress were reported in 24.5%, 35.9% and 33.3% of the cases, respectively. In addition, 13.9% of the subjects seemed to be at risk of developing PTSD [18].

To better understand the mental health burden of the COVID-19 pandemic among healthcare professionals, a validated measure of the pandemic-specific stressors is required. Such a measure may examine the stressors that are most burdensome and the way in which these stressors impact well-being and mental health. During the COVID-19 pandemic (Table 1), several measures were developed to capture distress or anxiety, sleep problems, or post-traumatic stress symptoms in the population, such as the *COVID Stress Scales* (CSSs) [22]; the *COVID-19 Fear Scale* (FCV-19S) [23]; the *Coronavirus Anxiety Scale* (CAS) [24], and the *COVID-19 Burnout Scale* (COVID-19 BS) [25]. These measures assess the perceived global level of stress during the COVID-19 pandemic but do not distinguish between different stressors, especially individual stressors, such as anxiety and fear for the health of oneself, family or colleagues, and organizational stressors, such as lack of proper communications and work overload. Recently, new measures have been developed to assess different stressors related to the pandemic, such as the *Pandemic Stress Questionnaire* (PSQ) [26]; the *COVID-19 Stressors Scale* [27]; the *COVID-19 Stress Scale* (CSS) [28]; the *COVID-19 Stressors Score* [29]; and the *Pandemic Stressor Scale* (PSS) [30] (Table 1).

Nevertheless, none of these measures have been built on the specific target of health workers despite being a population that was significantly affected by the COVID-19 pandemic, experiencing high levels of stress both on an individual (anxiety about health) and organizational level (lack of support and adequate communication in excessive workload) [18]. Starting from these premises, the goal of the present work was to develop a new scale to measure a broad range of different kind of stressors in healthcare operators during a highly critical and emergency period, such as that of the COVID-19 pandemic. To assess the multiple stressors of a pandemic or epidemic, it is necessary to have a valid and reliable measuring instrument that can capture as many as possible aspects that can induce high levels of stress. In order to construct the scale, we organized an integrated research project aimed specifically at developing such a measurement tool as one of the actions conducted by the Regional Reference Center on Relational Criticalities (RCRC), with Careggi University Hospital of Florence (Italy) as leading center, and it is based on the scientific collaboration with the Psychometric Lab of the NEUROFARBA (Neuroscience, Psychology, Drug Research and Child Health) Department of the University of Florence. Since its foundation in 2007 (DGR n.356/2007), the RCRC deals with the organizational well-being of hospitals and structures of the Regional Health System of Tuscany, promoting the psychological well-being of health care professionals through research, planning, consultancy and training. To reach the goal, a two-waves study, i.e., at T0, from June to September 2020, and at T1, from February to May 2021 (see Table 2 for the descriptive across the studies), was implemented.

We began the study in June 2020 during the first phase of the pandemic when healthcare professionals were involved at the forefront of the emergency management. In light of the limitations of the scales internationally generated during this period to assess psychological distress symptoms, the instrument that we present in the current paper was properly developed to measure distress derived from different individual and organizational stressors relevant during a pandemic or epidemic. This was done such that it could represent a useful tool to be used in possible future pandemic situations. For this reason, we named the scale *Individual and Organization-related Stressors in Pandemic Scale for Healthcare Workers* (IOSPS-HW). The goal was to build a new tool that allows to consider both the individual factors related to the health and well-being of individuals, such as family and personal relationships, fear of infecting others, difficulty in family management, uncertainty about the future and general disturbance of life, and organizational factors related to the management of the pandemic, such as workplace relationships, fear of infection, job management and communication, limited access to resources, and difficult working conditions. The construction of this new scale in the COVID-19 era highlighted the strong need to assess the severity of distress for different stressors relevant during a pandemic or epidemic in this category of workers. Combining aspects of individual and organizational stressors in a single tool that is valid and reliable might facilitate the implementation of actions with the purpose of reducing individual stress on the one hand and increasing organizational efficiency in emergency conditions on the other. In sum, the measurement of stress and its factors is essential to recognize more of the at risk groups of workers to prevent extremely stressful situations and perform adequate interventions. Given that there are no existing instruments created to comprehensively measure stressors during a pandemic among healthcare operators, the purpose of the present work was to develop a two-dimension and brief instrument to measure both individual and organizational factors. Indeed, we aimed to provide a scale appropriate for large, multivariate studies wherein several tests and scales must be administered together. In Study 1, we described the construction of the scale and its psychometric properties, in particular the analysis of its dimensionality. Study 2 offered a contribution to the measurement of its validity and reliability.

## 2. Study 1

The aim of Study 1 was to construct the scale and examine its psychometric properties through a cross-sectional design. Development of the IOSPS-HW began with the preparation of detailed construct specifications describing various aspects of stressors. In order to identify the macro areas from which to derive the potentially stressful factors for healthcare professionals in pandemic management, we analyzed the existing studies concerning previous pandemics (due to pathogens other than SARS-CoV2) [31] as well as the recommendations and guidelines published by international organizations, such as the World Health Organization (WHO) and Occupational Safety and Health Administration (OSHA) on the management of COVID-19 [32]. From such analysis, several factors, both related to individual health and organizational themes, emerged as potentially stressful. Following an initial examination of all these contributions, a first set of items was created to be submitted to a panel of healthcare workers based in the COVID-19 departments (doctors, nurses, psychologists, physiotherapists and social health workers) who analyzed the content and wording of the items. These specifications guided the creation of 43 candidate items with a 5-point Likert scale from 1 (*Not stressful*) to 5 (*Very stressful*). After this first phase, the scale was administered to a sample of healthcare professionals with the aim of studying dimensionality. We hypothesized that, in order to obtain the scale, we would need to assess two correlated dimensions measuring individual and organizational stress factors. In other terms, we expected to have a scale with a two-factor structure. In this way, we could obtain a single score for each dimension. Moreover, the benefit of a bi-correlated-factor construct is that scoring is simplified as items are added for the whole domain to achieve the total score.

We also assessed the reliability with the omega coefficient for each dimension and for the total scale, and the validity by examining the correlation with the post-traumatic level of stress [33]. In line with previous studies, we expected to find a positive correlation between work stress and distress experienced [18,34]. Finally, we investigated differences in the IOSPS-HW subscales and total scores by considering the work experience in a COVID-19 Unit, in line with other studies that have found substantially higher levels of stress among those healthcare operators who worked in COVID-19 units compared to those who did not work in COVID-19 units [17,21,35].

### 2.1. Participants

The sample was composed of 950 health workers (77% females) aged from 23 to 68 years (mean age = 47.76 years, *SD* = 10.20) recruited in the hospital of Careggi, Tuscany, in the period immediately following the first lockdown (from June to September 2020, T0). Table 2 depicts the socio-demographic variables investigated, i.e., marital status, level of education, type of job, and length of service. In addition, each participant was asked: “Did you work/do you work in a COVID-19 Unit?” Thirty-six percent of participants (*n* = 346) worked in a COVID-19 Unit.

### 2.2. Measures and Procedure

The research protocol included a form for gathering socio-demographic data (age, sex, marital status, educational qualification, professional qualification, and years of service). Additionally, it was requested to indicate whether the participants worked or had worked in a COVID-19 Unit.

The *Individual and Organization related Stressors in Pandemic Scale for Healthcare Workers* (IOSPS-HW) administered in Study 1 consisted of 43 items describing two areas of stressors: One related to individual health (19 items) and the other to organizational themes (24 items). Item examples were “Fear of getting severely ill” and “Work in solitude”, respectively. Items were rated on a 5-point scale ranging from 1 (*Not stressful*) to 5 (*Very stressful*) (see Appendix A).

To measure post-traumatic stress symptoms, the *Impact of Event Scale-Revised* (IES-R) [36,37] was used. The IES-R is a 22-item self-report evaluation scale to assess current subjective distress for a specific traumatic life event. Participants responded by considering a specific stressful life event while managing the COVID-19 health emergency and then indicating the level of distress they experienced in the past two weeks. The IES-R is composed of three subscales: Intrusions (e.g., repeated thoughts about the trauma), Avoidance (e.g., effortful avoidance of situations that serve as reminders of the trauma), and physiological hyperarousal. The IES-R score is defined as the sum of the average of the three subscales by providing an indication of the level of distress experienced, with a higher score indicating a greater psychological impact.

Participants filled out the research protocol online. The inclusion criterion included being a health worker actively working in the hospital during the epidemic’s first lockdown. Interested health workers were able to access the survey only after signing the informed consent form. Anonymity was preserved and the median survey completion time was approximately 30 min.

This study was not preregistered. Data will be available under request and after the permission of the Hospital.

### 2.3. Results

First, a missing analysis was conducted in order to identify cases with more than 10% of missing values. No such case was found. For cases with less than 10% of missing values, missing was replaced with the mean value of the total sample. This replacement was conducted only for 2% of the participants (*n* = 19).

#### 2.3.1. Item Selection and Dimensionality

The following process was used to reduce the original 43-item questionnaire to a shortened version. Item distributions and descriptions were examined for assessment of normality. Skewness and Kurtosis indices revealed that the departures from normality were acceptable [38]. An Exploratory Factor Analysis (EFA) with the Principal Component Analysis (PCA) with Oblimin rotation was performed, and two components were extracted. Results showed that eight items had factor loadings on both components (e.g., “*Discomfort due to work wearing individual safety devices (e.g., masks, visors, overalls*”) For this reason, these items were eliminated.

An EFA with the Principal Axis Factoring (PAF) and an Oblimin rotation was also performed on the resulting 35 items since the potential dimensions were expected to be correlated. The factorability was supported by Bartlett’s test of sphericity (*χ*^2^ = 22,219.32, *df* = 595, *p* < 0.001) and the Kaiser-Meyer-Olkin measure of sampling adequacy (0.95) [39]. The resulting two factors were defined as Individual- and Health-related Stressors (IH-S; 16 items) and Organization-related Stressors (O–S; 19 items). Results showed two correlated factors (*r* = 0.63). All items possessed factor loadings greater than 0.40, ranging from 0.40 to 0.78 on the expected factor.

Subsequently, the two-factor structure was tested by a confirmatory factor analysis (CFA) that employed the maximum likelihood estimator (AMOS software) [40]. To verify the model’s fit, the following indices were taken into account: The comparative fit index (CFI) [41], the Tucker–Lewis’s index (TLI) [42], and the root mean square error of approximation (RMSEA) [43]. For the TLI and CFI, values above 0.90 are indicative of acceptable fit, while values above 0.95 were indicative of excellent fit [44]. The RMSEA value is considered acceptable when it is below 0.08 and good when it is below 0.05 [45].

**Figure 1:**
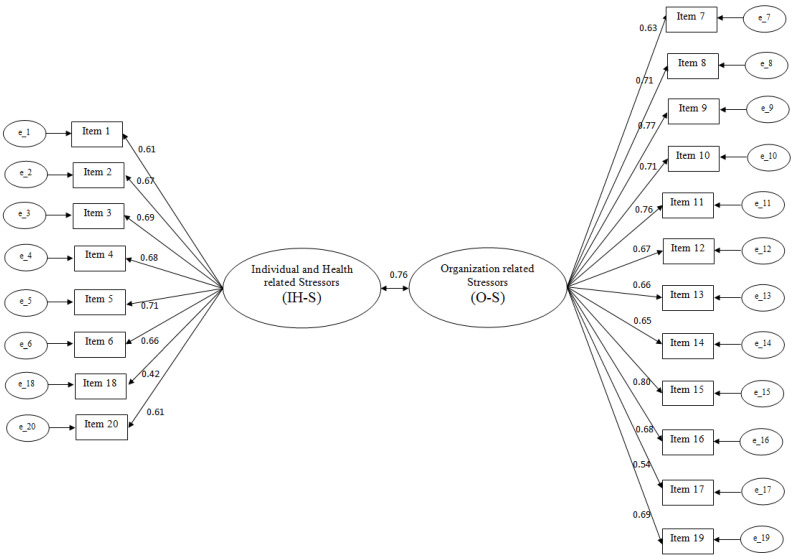
The two-factor model of the Individual- and Organization-related Stressors in Pandemic Scale for Healthcare Workers (IOSPS-HW) in T0 sample (*n* = 950).

The results showed a poor overall fit (TLI = 0.713, CFI = 0.746, RMSEA = 0.097, 90% CI [0.095, 0.100]). Modification indices (MIs) suggested adding error covariance between several items. Scrutiny of the content of items revealed a similarity in item content that may have led to error covariances [46]. Following the analysis of the factor loading of each item with the expected factor, we selected the items with the highest factor loading [47]. In this way, we eliminated 15 items. For example, between the item “*Excessive circulation of information/communications*” and the item “*Difficulty in identifying suitable interlocutors according to needs*” that had error covariance, we kept the second because it had a higher factor loading. The final version of the scale resulted in 20 items: Twelve items for Organization-related Stressors (O–S) and eight items for Individual- and Health-related Stressors (IH–S) (see Appendix A). This form demonstrated a good fit (TLI = 0.902, CFI = 0.913, RMSEA = 0.069, 90% CI [0.064, 0.073]). Each item loaded strongly and significantly on its hypothesized factor with factor loadings ranging from 0.42 to 0.77. The correlation between the factors was found to be 0.76 (Figure 1).

#### 2.3.2. Reliability and Validity

The omega value for the overall scale was 0.93 (95% CI [0.92, 0.93]) and all the corrected item-total correlations were above 0.30, ranging from 0.33 to 0.60. The value of omega did not increase if an item was deleted. The omega was 0.91 (95% CI [0.90, 0.92]) for the *Organization-related Stressors* (O–S) subscale, and 0.84 (95% CI [0.82, 0.85]) for the *Individual and Health related Stressors* (IH–S) subscale. Following the cut-offs proposed by the European Federation of Psychological Assessment (EFPA) [48], the values of internal consistency were good for the scale and the subscales.

With respect to validity, in order to investigate the relationships between organizational and individual stressors and post-traumatic stress, the correlations between the IOSPS-HW score and its subscale scores, as well as the IES-R, were calculated. The results illustrated that a high perception of organizational and individual factors as stressors was associated with a high perception of post-traumatic stress. In detail, the total score for the IOSPS-HW was significantly and positively correlated with the total score at the IES-R. Both the subscale scores were correlated with the total score at IES-R (Table 3).

All the correlation values were adequate or good measures of validity according to the cut-offs proposed by the EFPA [48]. Specifically, for criterion-related validity, values between 0.20 and 0.35 are deemed adequate, values between 0.35 and 0.50 are good, and values higher than 0.50 are excellent. To further address the validity, we assessed work environment differences in the IOSPS-HW score. Participants were divided into two groups according to their work unit (No-COVID-19 Unit vs. COVID-19 Unit). Independent sample *t*-tests were performed to explore the differences between the groups on the IOSPS-HW total score and each subscale. As hypothesized and reported in Table 4, participants who worked or had worked in a COVID-19 Unit (72% females; mean age = 44.78, *SD* = 10.27) demonstrated significantly higher scores on the total score and on each subscale compared to the participants who did not work in a COVID-19 Unit (79% females; mean age = 49.47, *SD* = 9.76).

### 2.4. Discussion

The aim of this study was to develop a new scale to measure organizational and individual stressors during a pandemic in healthcare operators. Our findings indicate that the IOSPS-HW is a two-factor structure assessing individual and organizational factors related to perceived stress. This scale might allow capturing a broader range of stressor domains. A strength of this study was the use of large-sized samples along with the combined use of EFA and CFA. The developed scale was confirmed to have convergent validity with an existing post-traumatic stress measure. Indeed, the IOSPS-HW score and the subscales significantly and positively correlated with the IES-R. Additionally, the IOSPS-HW was able to discriminate against health workers considering their unit. As expected, health workers who worked in COVID-19 units had a higher level of stress in each subscale and in the total score. Additionally, the IOSPS-HW presents all the advantages of the short scale and resulted to be appropriate for large multivariate studies where several measures must be administered together. In summary, it could be used to identify individuals who require additional psychological support. Nevertheless, this study had the limitation to be cross-sectional by involving a sample of healthcare operators during the first lockdown of the emergency in Italy. Thus, generalizability to other times of the pandemic is limited. For this reason, we conducted Study 2 to investigate the psychometric properties of the IOSPS-HW in a different wave of the pandemic.

## 3. Study 2

Following a second wave of infections occurring between September and December 2020, leading to the health situation being critical, a high number of infections, mortality rate and hospitalizations forced the hospitals to open numerous COVID-19 units. In the months of February to May 2021, there was a stabilization of the positivity and mortality rate and the number of hospitalizations. A characterizing aspect of this period was concerned with the introduction of anti-COVID-19 vaccines. Beginning on 27 December 2020 (“Vaccine Day”), the vaccination campaign began in Italy, which established the priority in the administration of vaccines to certain categories, including over 80, fragile categories, and health professions. In the following months from February to May 2021, the administration of the first dose of the vaccine began, and in the same months, the vaccination obligation for all those who practiced a health profession took over.

Starting from these considerations, the first aim of Study 2 was to confirm the factor structure of the final 20-item IOSPS-HW across various waves of the pandemic through a multigroup confirmatory procedure. Thus, we followed a longitudinal design. Additionally, we further tested the validity of the scale by taking into account depression, anxiety, well-being and post-traumatic stress as criterion variables by considering them indicators of the healthcare workers’ mental health [49]. Moreover, we investigated the predictive validity of the IOSPS-HW by testing the predictive power of each dimension at T0 with PTSD symptoms at T1. In this way, the weight of each dimension related to individual and organization stressors offering important support in the assessment for future crisis intervention for healthcare workers was tested. Finally, we provided further evidence for the reliability of the scale in terms of temporal stability.

### 3.1. Participants

Eight hundred and forty-three health workers (76% females) aged 22 to 69 years (mean age = 47.91 years, *SD* = 10.66) recruited in the hospital of Careggi, Tuscany, participated in a second survey (T1) that took place between February and May 2021. Table 2 depicts the socio-demographic variables for the time of administration, i.e., marital status, level of education, type of job, and length of service. In addition, each participant was asked: “Did you work/do you work in a COVID-19 unit?”. Thirty-eight percent of participants (*n* = 322) worked in a COVID-19 Unit.Among the participants, 522 cases (*M*_age_(*SD*) = 47.00 (11.09), 74% female) participated only in the second survey, and 321 (*M*_age_(*SD*) = 49.38 (9.78), 80% female) participated in both the waves (T0 and T1).

### 3.2. Measures and Procedure

As in Study 1, the research protocol included a form for gathering socio-demographic data (age, sex, marital status, educational qualification, professional qualification, and years of service) and participants were requested to indicate if they worked or had worked in a COVID-19 Unit. Participants then answered the IOSPS-HW and the IES-R, as in Study 1. However, in this study, the IOSPS-HW was the 20-item version with twelve items for the *Organization-related Stressors* (O-S) and eight items for the *Individual and Health related Stressors* (IH-S). Additionally, the *Generalized Anxiety Disorder-7* (GAD-7) [50,51], the *Patient Health Questionnaire* (PHQ-9) [51,52], and the *World Health Organization Well-being Index* (WHO-5) [53,54] were used to assess the criterion validity variables.

The GAD-7 [50,51] is a 7-item questionnaire developed to identify probable cases of generalized anxiety disorder and to measure the severity of symptoms based on the DSM-IV [55] such as nervousness, inability to stop worrying, excessive worry, and restlessness. The GAD-7 asks participants to rate how often they have been concerned by seven core symptoms over the past two weeks. Response categories are *not at all*, *several days*, *more than half the days*, and *nearly every day*, scored as 0, 1, 2, and 3, respectively. The total score of the GAD-7 ranges from 0 to 21. Among primary care patients and the general population, the GAD-7 has demonstrated good internal consistency, test-retest reliability, and convergent, construct, criterion, and factorial validity [50,52,56].

The PHQ-9 [51,52] is a self-report measure consisting of nine questions based on the nine DSM-IV criteria for a major depressive episode [55]. It refers to symptoms experienced during the two weeks prior to answering the questionnaires. Scores for each item range from 0 (*not at all*), to 1 (*several days*), 2 (*more than half of the days*) and 3 (*nearly every day*), while summed scores range from 0 to 27. The PHQ-9 can be used as a screening tool with recommended cut-off scores of ten or greater for the diagnosis of major depression.

The WHO-5 [51,52,53] allows for a brief assessment of well-being over a two-week period. Subjective well-being refers to how people experience and evaluate their lives. Individuals were asked to indicate for each of the five statements how they felt over the past two weeks, using a six-point Likert scale ranging from 0 (*at no time*) to 5 (*all of the time*). The scale has shown good psychometric properties in both general and clinical populations [57].

As in Study 1, participants filled out the questionnaire online and inclusion criterion was being a health worker actively working in the hospital during the epidemic. Anonymity was preserved and the median survey completion time was approximately 30 min.

### 3.3. Results

Similar to Study 1, we checked for missing data. However, no cases with more than 10 of the missing value were found. For cases with less than 10% of missing values, the missing value was replaced with the mean value of the total sample. This replacement was conducted only for 2% of the participants (*n* = 17).

As reported in Table 2, 42% of participants who took part in T1 worked in a COVID-19 Unit. Due to the health emergency, 35% of health workers were assigned to a different unit from the one in which they usually worked.

#### 3.3.1. Validity across Time

Invariance of the IOSPS-HW was tested by comparing the T0 sample, including all the subjects who participated only in the first survey (*n* = 617, *M*_age_(*SD*) = 47.32(10.35), 76% female) to the T1 sample, including all the subjects who participated only in the second survey (*n* = 522, *M*_age_(*SD*) = 47.00(11.09), 74% female). First, the two-factor model was tested separately in the two groups, and the model showed acceptable fit indices among T0 sample (TLI = 0.900; CFI = 0.901; RMSEA = 0.073, 90% CI [0.068, 0.079]), with standardized factor loadings ranging from 0.43 to 0.76 and two correlated factors (*r* = 0.74, *p* < 0.001). A good fit was also obtained for T1 sample (TLI = 0.914; CFI = 0.924; RMSEA = 0.066, 90% CI [0.060, 0.072]). The standardized factor loadings ranged from 0.51 to 0.81 and the correlation between the two factors was 0.71 (*p* < 0.001).

To test for cross-validation with a multigroup confirmatory analysis on the two-waves samples, analyses were conducted by performing hierarchically nested confirmatory factor analyses and invariance was evaluated using the criteria of ∆CFI less than 0.01 and the equivalent cut-off of 0.015 for RMSEA [46,58]. Following the guidelines for testing measurement invariance, the preliminary independence model was fitted (*χ*^2^ = 11,047.70, *df* = 380, *p* < 0.001) [59]. Thereafter, the configural invariance was established (CFI = 0.912, RMSEA = 0.050), and metric invariance was assessed (Table 5). Although the ΔCFI criterion was met (.001), further levels of structural variances and covariances, as well as measurement error variances and covariances were supported by referring to the same criterion (respectively 0.000 and 0.004).

#### 3.3.2. Criterion Validity

In order to analyze the criterion validity of the IOSPS-HW, we investigated its associations with depression, anxiety, well-being and post-traumatic stress. In line with other studies, a positive correlation was found between the total and subscale scores at the IOSPS-HW with depression, general anxiety and post-traumatic stress studies [18,60,61]. Moreover, a negative correlation was found between the IOSPS-HW total and subscale scores with well-being (Table 6).

#### 3.3.3. Predictive Validity 

We also assessed the predictive validity of the IOSPS-HW scale by investigating its predictive power on PTSD symptoms by considering the participants in both the waves. In particular, a stepwise linear regression analysis was run with PTSD symptoms as the dependent variable and the IOSPS-HW subscale scores as the independent variables. Preliminarily, we verified that the change of the rate of infections between T0 (2.18%) and T1 (8.41%), i.e., +6.23%, was not significantly related to PTSD symptoms (*β* = 0.01, *p* = 0.872).

Thus, we included only the IOSPS-HW subscale scores in the regression analysis. In the first step (Model 1), only the *Individual and Health related Stressors* (IH–S) score was entered, and it was a significant and positive predictor. In Model 2, the *Organization-related Stressors* (O-S) was also introduced, which depicted a significant increase in explained variance (ΔR = 0.03) (Table 7).

#### 3.3.4. Temporal Stability

In order to analyze the reliability of the scale we measured the test-retest correlation (*n* = 297) for each dimension and the total score by considering the scores at T0 and T1. We obtained a coefficient of 0.67 (*p* < 0.001) for *Individual and Health related Stressors* (IH-S), 0.65 (*p* < 0.001) for *Organization-related Stressors*(O–S), and 0.70 (*p* < 0.001) for the IOSPS-HW total score scale. In line with the criteria for the EFPA, the scale showed good stability with correlations that were higher than 0.60 [48].

### 3.4. Discussion

In Study 2, the psychometric properties of the IOSPS-HW in a different wave of the pandemic were analyzed. This analysis was necessary as the scale was developed at a time when the response capacity of professionals was under threat, as they were insufficient resources to care for COVID-19 patients, contradictory instructions, or interruptions in the continuity of care of non-COVID-19 patients. Results confirmed the structural validity of the scale as it was found to be invariant across time. As expected, we found positive correlations between individual and organizational factors with anxiety, depression and post-traumatic symptoms and negative correlations with well-being. The predictive validity of the scale was also confirmed. To summarize, the IOSPS-HW scale resulted to be a valid tool to monitor stressor levels in order to check on the effective recovery of health care professionals.

## 4. Conclusions

During previous pandemics (e.g., Severe Acute Respiratory Syndrome (SARS), influenza A/H1N1, and the Middle East Respiratory Syndrome (MERS)), healthcare workers experienced severe problems such as emotional stress, including anxiety, depressive symptoms, and insomnia [14,62,63,64]. However, with respect to recent previous cases, the COVID-19 pandemic has been found to be more global and temporarily more widespread. The construction of this new scale in the COVID-19 era responds to the strong need to assess the severity of distress for different stressors relevant during a pandemic or epidemic in this category of workers. Through the development of this scale, our goal was to establish an evidence-based assessment that could be used as a basis for future crisis intervention for healthcare workers during any infectious disease outbreak. Combining aspects of the individual and organizational stressors in a single tool that is both valid and reliable may facilitate the implementation of actions that have the purpose of reducing individual stress on the one hand and increasing organizational efficiency in emergency conditions on the other. In summary, the measurement of stress and its factors is essential to recognize groups that are more at risk to prevent extremely stressful situations and carry out adequate interventions. This information is crucial to deploy targeted interventions to support professionals in terms of individual, group and organizational support. Specifically, thanks to the information obtained from the tool, information and psychoeducational materials can be prepared to help professionals recognize the signs of stress, and if necessary, ask for specialist help. In addition, initiatives can be arranged to support team working, and psychological support services can be strengthened. In line with the findings, more extensive training can be carried out for newly hired workers.

Through future surveys, the employment of the scale will allow to monitor any changes over time in the factors that may determine a source of stress for healthcare professionals who are managing various phases of the pandemic trend, thus guiding support actions. Overall, the emerging results and the experience gained could have significant consequences in terms of planning further actions aimed at mitigating the impact both on health professionals, and by extension, on the general population.

The IOSPS-HW has the valuable advantage of providing two separate scores for individual and organizational stress factors, compared to all the scales that have been built during the pandemic that do not separate these two dimensions. This is important because the literature suggests that healthcare workers experience high levels of stress which negatively affect their well-being. Moreover, research indicates that high levels of stress and low levels of well-being negatively affect productivity, that is a fundamental aspect for hospitals as the main providers of public health. The drop in productivity could lead to an increase in demand, with repercussions on levels of organizational stress and therefore healthcare workers’ well-being. Finally, the advantage of having the possibility of separately monitoring individual and organizational stressors allows for the structuring of diversified interventions which aim to work on different aspects. Other values of the IOSPS-HW regard the fact that it can be useful to monitor healthcare workers’ stress levels in other future possible pandemics. Indeed, the items do not refer specifically to the COVID-19 pandemic-contrary to some scales built during the pandemic itself.

This study had a few limitations. The first limitation is that it does not discriminate between professional categories, nor does it consider critical services separately during this crisis, such as critical care and resuscitation, internal medicine, pneumology, and infectious diseases. Moreover, these data are limited to Italy. A measurement of the invariance among languages could be conducted in future studies. Despite the limitations, the IOSPS-HW scale could be used in future outbreaks to check the level of acute stress of the health care workforce and to give them proportional support according to their needs in these conditions.

## Figures and Tables

**Table 1:** Measures of distress, anxiety, sleep problems, or post-traumatic stress symptoms during the COVID-19 pandemic in chronological order of publication.

Authors, Country	Scale (Number of Items)	Sample (*n*)	Cronbach’s Alpha	Factor Structure	Dimensions	EFA/CFA	Validity Measures
Ahorsu et al., 2020 [23], Iran	*The Fear of COVID-19 Scale* (7 items)	Iranian adults (717)	0.82	Unidimensional structure	Fear of COVID-19	EFA	depression, anxiety, perceived infectability and germ aversion
Lee, 2020 [24], US	*Coronavirus Anxiety Scale* (5 items)	US adults (775)	0.93	Unidimensional structure	Coronavirus anxiety	EFA and CFA	Coronavirus diagnosis, impairment, alcohol/drug coping, negative religious coping, extreme hopelessness, suicidal ideation
Taylor et al., 2020 [22], Canada and US	*COVID Stress Scales* (30 items)	US (3375) and Canadian (3479) adults	From 0.83 to 0.95	5-factor structure	COVID danger and contamination fears, COVID fears about economic consequences, COVID xenophobia, COVID compulsive checking and reassurance seeking, and COVID traumatic stress symptoms.	EFA (Canadian sample), CFA (US sample)	health anxiety, obsessive-compulsive (OC) contamination, checking symptoms, social desirability
Park et al., 2020 [27], USA	*COVID-19 Stressors* (23 items)	USAdults (1015)	-	Three subscales	Infection-related stressors, Daily activity stressors and Financial/resource-related efforts	-	-
Yıldırım and Solmaz, 2020 [25], Turkey	*COVID-19 Burnout Scale* (10 items)	Turkish adults (402)	0.92	Unidimensional structure	COVID-19 Burnout	EFA, CFA	COVID-19 stress and resilience
Ettman et al., 2020 [29], US	*COVID-19 Stressors Score* (13 items)	US adults (1441)	-	-	-	-	Depression symptoms
Kujawa et al., 2020 [26], US	*Pandemic Stress Questionnaire* (25 items)	US emerging adults (450)	From 0.72 to 0.79	Six subscales	General Perturbation of Life, Interpersonal, Financial, Educational/Professional Goals, Health-self and Health-others	-	General stress
Ahuja, 2021 [28], India	*COVID-19 Stress Scale* (21 items)	Indian adults (1009)	From 0.69 to.85	5-factor structure	Vexation with Others, Immediate Concerns, Routine Disruption, Uncertainty about the Future, and Systemic stressors	EFA	Coping strategies and COVID-19 stress
Lotzin et al., 2022 [30], Germany and Austria	*Pandemic Stressor Scale* (30 items)	Germany (2760) and Austrian (1021) adults	from 0.63 to 0.94	9-factor structure	‘Restricted Face-to-Face Contact’, ’Problems with childcare’, ‘Work-related problems’, ‘Fear of infection’, ‘Burden of infection’, ‘Restricted activity’, ‘Crisis management and communication’, ‘Restricted access to resources’, and ‘Difficult housing condition’	EFA, CFA	-

Note. EFA = Exploratory Factor Analysis, CFA = Confirmatory Factor Analysis.

**Table 2:** Socio-demographic variables of three groups of participants in two different surveys (T0 and T1).

Socio-Demographic Variables		Study 1	Study 2
		T0 (*n* = 950)	T1 (*n* = 843)
		*M* (*SD*)	*M* (*SD*)
Age		47.76 (10.20)	47.91 (10.66)
		Frequency (%)	Frequency (%)
Sex	Male	222 (23)	199 (24)
	Female	728 (77)	644 (76)
Marital status	Unmarried	258 (27)	244 (29)
	Married	456 (48)	385 (46)
	Divorced	52 (6)	54 (6)
	Separated	49 (5)	43 (5)
	Widower	19 (2)	10 (1)
	Cohabitant	116 (12)	107 (13)
Educational qualification	Middle/high school diploma	264 (28)	270 (32)
	Bachelor’sdegree	332 (35)	297 (35)
	Master’sdegree	139 (15)	93 (11)
	Post-graduate qualifications/doctorate	215 (22)	183 (22)
Professional qualification	Doctor	206 (22)	160 (19)
	Nurse	415 (44)	409 (49)
	SSO	106 (11)	112 (13)
	Healthtechnician	90 (9)	74 (9)
	Other	133 (14)	88 (10)
Years of service	Lessthan 12 months	74 (8)	88 (10)
	From 1 to 2 years	540 (6)	43 (5)
	From 2 to 5 years	84 (9)	93 (11)
	From 5 to 10 years	70 (7)	44 (5)
	From 10 to 15 years	144 (15)	115 (14)
	From 15 to 20 years	156 (16)	111 (13)
	Over 20 years	368 (38)	349 (42)

**Table 3:** Pearson’s correlations between the Impact Events Scale-Revised (IES-R) score and the Individual- and Organization-related Stressors in Pandemic Scale for Healthcare Workers (IOSPS-HW) subscales and total scores.

	1.	2.	3.	4.	*M*	*SD*
IES-R score	-				49.39	17.85
2. *Individual and Health related Stressors (IH-S)*	0.64 ***	-			23.84	7.58
3. *Organization-related Stressors (O-S)*	0.52 ***	0.69 ***	-		38.24	11.42
4. *IOSP-HW*—Total score	0.61 ***	0.88 ***	0.95 ***	-	62.08	17.50

Note: *** *p* < 0.001; Impact Events Scale-Revised (IES-R); Individual- and Organization-related Stressors in Pandemic Scale for Healthcare Workers (IOSPS-HW).

**Table 4:** Differences between participants who did not work in COVID Units and participants who worked/had worked in COVID Units for the *Individual and Organization related Stressors in Pandemic Scale for Healthcare Workers* (IOSPS-HW) subscales and total score.

	*N*	*M*	*SD*	*t*	*df*	*p*	Cohen’s *d*
*Individual and Health related Stressors (IH-S)*							
COVID Unit	346	25.21	7.21				
NO-COVID Unit	604	23.06	7.68	4.25	948	<0.001	0.29
*Organization-related Stressors (O-S)*							
COVID Unit	346	40.18	11.23				
NO-COVID Unit	604	37.13	11.39	3.99	948	<0.001	0.27
*IOSPS-HW*—Total score							
COVID Unit	346	65.39	16.74				
NO-COVID Unit	604	60.19	17.66	4.45	948	<0.001	0.30

Note: Individual- and Organization-related Stressors in Pandemic Scale for Healthcare Workers (IOSPS-HW).

**Table 5:** Goodness-of-fit statistics for each level of structural and measurement invariance across T0 and T1 samples for the *Individual and Organization related Stressors in Pandemic Scale for Healthcare Workers* (IOSPS-HW).

	CFI	RMSEA	ΔCFI	ΔRMSEA
1. Invariance of model configuration (*Configural invariance*)	0.912	0.050	-	-
2. Invariance of factor loadings (*Weak or Metric invariance*)	0.911	0.048	0.001	0.002
3. Invariance of structural variances/covariances	0.911	0.048	0.000	0.000
4. Invariance of measurement error variances/covariances	0.907	0.048	0.004	0.000

Note. CFI = comparative fit index; RMSEA = root mean square error of approximation; ΔCFI = difference between CFIs; ΔRMSEA = difference between RMSEAs.

**Table 6:** Pearson correlations between the *Individual and Organization related Stressors in Pandemic Scale for Healthcare Workers* (IOSPS-HW) subscale and total scores, Well-being, Depression, Generalized Anxiety and PTSD symptoms.

	1	2	3	4	5	6	7	*M*	*SD*
Well-being	-							18.57	6.77
2. Depression	−0.69 ***	-						8.69	6.28
3. Anxiety	−0.67 ***	0.84 ***	-					8.53	5.83
4. PTSD Symptoms	−0.47 **	0.65 ***	0.72 ***	-				54.78	21.32
5. *Individual and Health related Stressors (IH-S)*	−0.37 ***	0.52 ***	0.57 ***	0.68 ***	-			24.83	7.19
6. *Organization-related Stressors (O-S)*	−0.33 ***	0.42 ***	0.45 ***	0.52 ***	0.65 ***	-		39.92	11.78
7. *IOSP-HW*—Total score	−0.38 ***	0.50 ***	0.54 ***	0.63 ***	0.85 ***	0.95 ***	-	64.74	17.32

Note: ** *p* < 0.01; *** *p* < 0.001; Individual- and Organization-related Stressors in Pandemic Scale for Healthcare Workers (IOSPS-HW).

**Table 7:** Hierarchical linear regression with *Individual and Organization related Stressors in Pandemic Scale for Healthcare Workers* (IOSPS-HW) subscale score at T0 as independent variables and PTSD symptoms at T1 as dependent variable.

Predictors	*B*	*β*	*p*	R^2^	Adj. R^2^	Models Comparison	ΔR^2^	*p*
Model 1 *				0.31	0.31	-	-	-
*Individual and Health related Stressors (IH-S)*	1.55	0.56	<0.001					
Model 2 **				0.34	0.33	Model 2–Model 1	0.03	<0.001
*Individual and Health related Stressors (IH-S)*	1.06	0.38	<0.001					
*Organization-related Stressors (O-S)*	0.46	0.25	<0.001					

Note. * *F*(1,285) = 126.57, *p* < 0.001; ** *F*(2,284) = 72.69, *p* < 0.001; *n* = 287.

## Data Availability

Data available on request due to restrictions eg ethical.

## Appendix A. Individual- and Organization-Related Stressors in Pandemic Scale for Health Workers (IOSPS-HW)—The Final Version (20 Items)

We are now presenting to you a series of possible situations that you may have experienced, both on a professional and personal level, since the beginning of the COVID-19 pandemic. We ask you to respond by indicating how much each of the situations represented a source of stress for you on a scale of 1 (*Not stressful*) to 5 (*Very stressful*).

1. Illness or deaths of colleagues [IH-S] *****	1	2	3	4	5
2. Fear for getting severely ill [IH-S]	1	2	3	4	5
3. Social stigma because potentially in contact with infected patients [IH-S]	1	2	3	4	5
4. Reduction of time for self-care (e.g., rest, nutrition, movement) [IH-S]	1	2	3	4	5
5. Worries for own children or other family members [IH-S]	1	2	3	4	5
6. Suffering due to ethical dilemmas [IH-S]	1	2	3	4	5
7. Lack of clarity of role and tasks to be performed [O-S] **	1	2	3	4	5
8. Work in solitude [O-S]	1	2	3	4	5
9. Excessive pace and workload [O-S]	1	2	3	4	5
10. Receiving conflicting information [O-S]	1	2	3	4	5
11. Low support from the Manager [O-S]	1	2	3	4	5
12. Rigidity of procedures [O-S]	1	2	3	4	5
13. Difficulties related to the supply of devices, materials and services [O-S]	1	2	3	4	5
14. Long shifts [O-S]	1	2	3	4	5
15. Scarce circulation of information/communications [O-S]	1	2	3	4	5
16. Difficulty in identifying suitable interlocutors according to needs [O-S]	1	2	3	4	5
17. Constant pressure and stress to keep high professional commitment [O-S]	1	2	3	4	5
18. Be/have been placed in solitary confinement as a precaution [IH-S]	1	2	3	4	5
19. Perception of working closely with other professionals not adequately trained in emergency management or new tasks [O-S]	1	2	3	4	5
20. Assistance to patients potentially positive for the infection [IH-S]	1	2	3	4	5
* [IH-S]: Individual- and Health-related Stressors; ** [O-S]: Organization-related Stressors.					
